# Supplementary material for: The genome sequence of the Grey Tortrix moth, Cnephasia stephensiana (Doubleday, 1850) (Lepidoptera: Tortricidae)
Source: Wellcome Open Res. 2025 Oct 6;10:547. [Version 1] doi: 10.12688/wellcomeopenres.24977.1 (PMC12775664; doi:10.12688/wellcomeopenres.24977.1)
Supplement: Peer review history

# Reviewer report: Reviewer response for version 1

**DOI:** 10.21956/wellcomeopenres.27512.r137067

**Published:** 2026-01-06

**Authors:** Xueyan Li

**Affiliations:** 1 Kunming Institute of Zoology, Chinese Academy of Sciences, Kunming, China

This paper “The genome sequence of the Grey Tortrix moth, *Cnephasia stephensiana* (Doubleday, 1850) (Lepidoptera: Tortricidae)” assembled the chromosomal-level genome of Tortrix moth, *Cnephasia stephensiana*. The high-quality genome assembly including two haplotypes will be helpful to investigate the evolution of moth diversity. In general, this paper is well written with concise description.

Are sufficient details of methods and materials provided to allow replication by others?

Yes

Is the rationale for creating the dataset(s) clearly described?

Yes

Are the datasets clearly presented in a useable and accessible format?

Yes

Are the protocols appropriate and is the work technically sound?

Yes

Reviewer Expertise:

Entomology

I confirm that I have read this submission and believe that I have an appropriate level of expertise to confirm that it is of an acceptable scientific standard.

# Reviewer report: Reviewer response for version 1

**DOI:** 10.21956/wellcomeopenres.27512.r140093

**Published:** 2025-12-29

**Authors:** Panagiotis Ioannidis

**Affiliations:** 1 Foundation for Research & Technology - Hellas, Crete, Greece

This paper describes the sequencing and assembly of the genome of Cnephasia stephensiana, a lepidopteran insect.

 Haplotype 1 of the assembly is of excellent quality since virtually all of its contigs are assigned to one of the 30 chromosome-level scaffolds. In agreement with the excellent contiguity, BUSCO scores (98.7% complete) and Merqury scores (QV = 65.7, 99.3% k-mer completeness) are also very good. Finally, the blobplot shows that virtually all the contigs have approximately 100x coverage and around 38% GC content. It would be nice if the authors could comment on the potential origin of the smaller contigs.

 As I have mentioned before, I believe that it is of the utmost importance to include a predicted gene set, in order to increase the utility of such genome assemblies. Of course, I noticed (in the last section) that the authors plan to provide a gene set via Ensembl, but it would be so much better if this gene set was provided together with this genome report.

Are sufficient details of methods and materials provided to allow replication by others?

Yes

Is the rationale for creating the dataset(s) clearly described?

Yes

Are the datasets clearly presented in a useable and accessible format?

Yes

Are the protocols appropriate and is the work technically sound?

Yes

Reviewer Expertise:

bioinformatics; insect genomics

I confirm that I have read this submission and believe that I have an appropriate level of expertise to confirm that it is of an acceptable scientific standard.
